# Structure-based discovery of selective vaccinia-related kinase 1 inhibitors and fluorogenic active-site probes

**DOI:** 10.1016/j.jbc.2026.111355

**Published:** 2026-03-06

**Authors:** Emma K. Crowley-Dolen, Rafael Junqueira Borges, Paul S. Charifson, N. Connor Payne, Ramon Guerra de Oliveira, Micael Rodrigues Cunha, Ralph Mazitschek, Katlin B. Massirer, Timothy J. Mitchison

**Affiliations:** 1 Program in Chemical Biology, Harvard University, Cambridge, Massachusetts, USA; 2 Therapeutics Graduate Program, Harvard Medical School, Boston, Massachusetts, USA; 3 Center for Molecular Biology and Genetic Engineering (CBMEG), Universidade Estadual de Campinas (UNICAMP), Campinas, São Paulo, Brazil; 4 Center of Medicinal Chemistry (CQMED), Structural Genomics Consortium (SGC), UNICAMP, Campinas, São Paulo, Brazil; 5 Harvard Medical School Therapeutics Translator, Blavatnik Institute, Harvard Medical School, Boston, Massachusetts, USA; 6 Department of Chemistry and Chemical Biology, Harvard University, Cambridge, Massachusetts, USA; 7 Center for Systems Biology, Massachusetts General Hospital, Boston, Massachusetts, USA; 8 Broad Institute of MIT and Harvard, Cambridge, Massachusetts, USA; 9 Department of Immunology and Infectious Diseases, Harvard T. H. Chan School of Public Health, Boston, Massachusetts, USA; 10 Department of Systems Biology, Harvard Medical School, Boston, Massachusetts, USA

**Keywords:** cancer, cancer biology, cancer therapy, docking, drug screening, enzyme inhibitor, fluorescence resonance energy transfer (FRET), inhibitor, serine/threonine protein kinase, virtual screening

## Abstract

Vaccinia-related kinase 1 (VRK1) is a promising therapeutic target in gliomas and glioblastomas where VRK2 is silenced by promoter methylation, rendering VRK1 essential for accurate nuclear envelope reassembly following mitosis. Small-molecule ATP-site drug discovery for VRK1 has been hindered by the absence of robust and reproducible biochemical assays. Through virtual screening, we identified previously unreported VRK1-binding scaffolds and validated them in biochemical kinase assays, yielding an 82 nM inhibitor with high selectivity for VRK1 over VRK2. During characterization of this compound, we found that a commonly used commercial time-resolved fluorescence resonance energy transfer VRK1 activity assay is dependent on purification tag-mediated VRK1 dimerization. Leveraging the new inhibitor, we developed fluorogenic tool compounds that increase in fluorescence intensity upon binding to the active site of VRK1 and do not require artificial dimerization of VRK1. The top probe exhibits a K_d_ of 180 nM and is useful for ligand displacement assays using both fluorescence enhancement and time-resolved fluorescence resonance energy transfer readouts. Together, these results introduce new chemical scaffolds for targeting VRK1, define an assay artifact that has complicated VRK1 inhibitor discovery, and deliver fluorogenic tool compounds for high-throughput screening of ATP-site VRK1 inhibitors, enabling future drug discovery efforts against this emerging cancer vulnerability.

The vaccinia-related kinase (VRK) family, named for its homology with the vaccinia virus B1 kinase, comprises three proteins in humans: VRK1, VRK2, and VRK3 (1, 2, 3). VRK1 and VRK2 are serine/threonine kinases, whereas VRK3 is believed to be a pseudokinase (1, 4). VRK1 and its invertebrate homologs bind to chromatin and regulate chromatin remodeling during mitotic entry and exit (5, 6, 7, 8, 9, 10, 11). VRK1 is also thought to modulate certain transcription factors and participate in the DNA damage response (9, 12, 13, 14). The best characterized VRK1 substrates include BAF (BANF1), which cross-links chromatin during nuclear envelope assembly (10, 15, 16), p53 (12, 17, 18, 19, 20), and VRK1 itself (12, 18, 20, 21, 22, 23, 24, 25, 26). Casein is also widely used as a model substrate in biochemical assays (1, 18). VRK2 is less well studied. It shares 44% overall sequence similarity with VRK1, rising to 53% within the kinase domains (1). VRK1 contains a nuclear localization signal and is primarily localized in nuclei, although some is cytosolic (1, 18, 19, 27, 28). VRK2, by contrast, exists as two isoforms: VRK2a, the full-length protein, contains a membrane-spanning region and associates with the endoplasmic reticulum. VRK2b, a truncated isoform lacking the C-terminal 111 amino acids, localizes to both the cytosol and nucleus (1, 17). Despite their differences in localization and structure, VRK1 and VRK2 share many of the same substrates and exhibit functional redundancy (15, 16, 17, 23, 24, 29, 30, 31).

Recently, VRK1 has emerged as a potential target for the treatment of various cancers. Elevated expression of VRK1 correlates with poor prognosis in glioma, neuroblastoma, hepatocellular carcinoma, and breast cancer (30, 31, 32, 33, 34, 35, 36, 37, 38). Furthermore, VRK1 is essential in cancers lacking VRK2, making it an attractive target for glioblastoma, a major unmet medical need. In approximately two-thirds of low-grade gliomas and glioblastomas, VRK2 is silenced by its promoter methylation, creating a dependency on VRK1 for cell survival (30, 31, 39, 40). In VRK2-deficient cells, genetic knockdown of VRK1 causes defects in nuclear reassembly following mitosis, which has been attributed to insufficient BAF phosphorylation during prophase (30, 31). These observations prompted efforts to identify selective small-molecule inhibitors of VRK1. The first resolved ligand co-crystallized with VRK1 was BI-D1870, an RSK inhibitor, which enabled structure-guided development of pteridinone-based inhibitors (41, 42). Optimization of this scaffold by varying three key substituents yielded VRK1/CK1-IN-1, a 38 nM VRK1 inhibitor with greater than 90-fold selectivity over VRK2 (42). However, this compound also inhibits CK1-δ and CK1-ε with low nanomolar potency. Beyond the pteridinone series, a pyridine-based compound has been reported as a 150 nM inhibitor with activity across the VRK family (43).

So far, most VRK1 inhibitor discovery efforts have focused on the pteridinone and pyridine scaffolds. Screening of focused kinase inhibitor libraries has yielded few additional chemotypes, likely because VRK1 possesses a unique αC4 helix between αC and β4, and shares less than 30% sequence identity with other CK1 kinase family members (4, 24, 41, 44). These features make VRK1 structurally distinct and difficult to target using inhibitors optimized for related kinases. Consequently, broader and more chemically diverse screening strategies are required to identify novel VRK1 inhibitors.

A bottleneck to VRK1 inhibitor discovery has been the absence of reliable high-throughput screening assays. Early efforts relied on differential scanning fluorimetry (41), which can detect ligand-induced protein stabilization, but does not provide quantitative binding affinities or dose-response relationships. Radiometric kinase activity assays using [γ-^32^P]ATP have been effective for benchmarking VRK1 activity across substrates, but are low throughput and inaccessible for many labs (7, 9, 16, 18, 20, 22, 23, 24, 26, 29, 45, 46). More recently, a commercial time-resolved fluorescence resonance energy transfer (TR-FRET) assay became the standard platform for VRK1 inhibitor screening (42, 43). This assay employs a histone H3 peptide labeled with a ULight fluorophore (Revvity cat # TRF0125). Phosphorylation of this peptide is recognized by a europium-chelated anti-phospho-H3 antibody, generating a FRET signal between the labeled peptide and detection antibody. Although quantitative and high-throughput, the commercial TR-FRET assay has important limitations. The ULight-H3 substrate is reported to support the measurement of both VRK1 and VRK2 activity, but kinase activity against this substrate is strongly influenced by the purification tag on the VRK1 or VRK2 construct (42, 43). Furthermore, the assay is performed near the edge of steady-state enzyme kinetics. Because the substrate is fluorescently labeled, increasing its concentration raises background signal. Thus, to increase specific signal from the phosphorylation of H3, either ATP or VRK1 concentration must be raised. Increasing ATP above the K_m_ makes screening ATP-site inhibitors difficult as weak inhibitors will be unable to compete with the high ATP concentration. Increasing VRK1 concentration moves the reaction out of the steady-state kinetics regime. Finally, the commercial TR-FRET assay is prohibitively expensive for large-scale compound screens.

In this study, we report the first large-scale, chemically diverse, structure-based virtual screening campaign for VRK1 inhibitors, which we further validate experimentally. This approach enables *in silico* evaluation of large libraries that would be time-consuming and costly to test experimentally. This virtual screen yielded a top compound with a K_i_ of 82 nM that is selective over VRK2. We then leveraged this scaffold to develop three active-site fluorescent probes for VRK1, enabling ligand-displacement assays with either fluorescence dequenching or TR-FRET readouts. These assays allow accurate measurement of inhibitor K_d_ values and support cost-efficient, high-throughput biochemical screening. Notably, inhibitor K_d_ values obtained using the probe displacement assay were unaffected by the purification tag on VRK1, whereas activity in the commercial TR-FRET assay (ULight-H3) was highly dependent on tag-mediated dimerization. These results indicate that the probe displacement assay circumvents artifacts in the commercial assay to provide a more robust and reliable platform for biochemical characterization of VRK1 inhibitors.

## Results

### Virtual screening for VRK1 inhibitors

**Figure 1:**
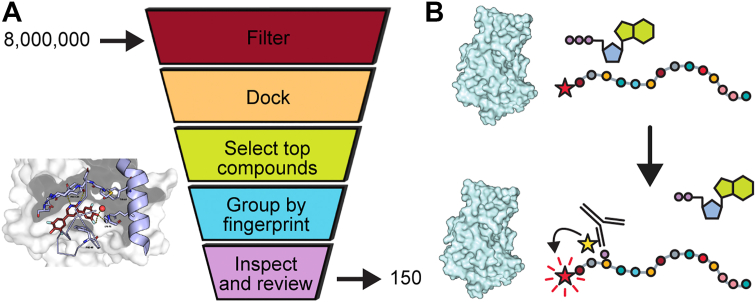
***In silico* to biochemical pipeline for the discovery of VRK1 inhibitors.***A*, docking pipeline used to determine which compounds to test *in vitro*: MolPort library of eight million in stock compounds was initially filtered for drug-like properties and possible PAINS compounds; remaining four million compounds were docked. Top compounds were selected and grouped by fingerprint interaction with VRK1, and top compounds from each fingerprint were reviewed by eye (see Experimental Procedures). *B*, modified commercial TR-FRET kinase activity assay used to test compounds for the inhibition of VRK1-mediated phosphorylation of histone H3 peptide. PAINS, pan-assay interference compound; VRK, vaccinia-related kinase; TR-FRET, time-resolved fluorescence resonance energy transfer.

To identify novel ATP-site ligands for VRK1, we first surveyed inhibitor-bound crystal structures of VRK1 that were suitable for computational docking. Review of published co-crystal structures of VRK1 bound to ATP-site inhibitors found in the Protein Data Bank (PDB) showed that a buried active-site water molecule frequently mediates hydrogen bond formation with ligands (41), (6VXU, 6CNX, 6CMM, 6BRU, 6DD4, 6CSW, 6CQH, 6BP0). Using a co-crystal structure of VRK1 bound to compound 26 (PDB 6BU6) (43), we generated two docking grids: one retaining the conserved water molecule to capture water-mediated interactions and one excluding it to identify ligands capable of displacing the water. Using these two docking grids, we virtually screened approximately four million compounds from the MolPort library. Hits were prioritized for predicted hinge binding to Phe134, filtered by strain energy (<4 kcal/mol), and clustered using protein–ligand interaction fingerprints to ensure chemical and interaction diversity. Representative high-scoring compounds from each cluster were visually inspected, yielding 150 candidates selected for biochemical testing against VRK1 (Fig. 1*A*). This dual-grid strategy enabled the identification of chemically diverse scaffolds capable of exploiting or displacing the conserved active-site water molecule, thereby broadening the range of potential VRK1 inhibitor chemotypes.

### Biochemical follow-up of virtual hits

We evaluated the inhibitory potency of compounds from the virtual screen using the commercial TR-FRET kinase activity assay. VRK1 was expressed in bacteria as an artificial dimer by fusion to GST, consistent with previous studies (42, 43). This dimeric construct was necessary to obtain measurable activity in the commercial TR-FRET assay, for reasons described below.

**Figure 2:**
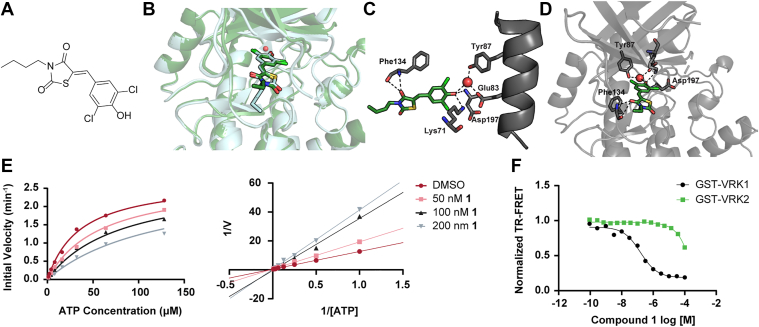
**Compound 1 is a potent VRK1 inhibitor, selective for VRK1 over VRK2.***A*, chemical structure of Compound 1. *B*, Compound 1 docked into PDB 6BU6 VRK1 (*teal*) overlaid with true co-crystal structure of Compound 1 bound to VRK1 (*green*). *C*–*D*, labeled interactions of co-crystallized Compound 1 (*green*) with VRK1 (*gray*). *E*, Compound 1 is an ATP-competitive VRK1 inhibitor. *F*, Compound 1 is selective for VRK1 over VRK2. VRK, vaccinia-related kinase.

GST-VRK1 was preincubated with test compounds and subsequently combined with ATP and histone H3 peptide conjugated to ULight fluorophore (acceptor). Phosphorylation of the peptide was detected using a europium-labeled anti-phospho-H3 antibody (donor) (Fig. 1*B*). Of the 150 compounds selected from the docking screen, nine had activity against VRK1 (defined as IC_50_ < 15 μM) and three displayed more than tenfold selectivity for VRK1 over VRK2 (Table S1 and Figs. S1 and S2). The most potent compound, Compound 1 (Fig. 2*A*), yielded an IC_50_ value of 154 nM (95% confidence interval of 96–245 nM) and was selective over VRK2 (Fig. 2*F*).

### Compound 1 is an ATP-competitive inhibitor

Because the virtual screen was designed to identify ATP-site binders, we next confirmed the mode of inhibition for Compound 1. We developed a variation of the commercial TR-FRET assay in which europium anti-pH3 antibody was replaced with a nonfluorescent rabbit IgG anti-pH3 antibody and a CoraFluor 1–conjugated anti-rabbit IgG nanobody. VRK1 was preincubated with 75 nM ULight-H3 peptide and increasing concentrations of Compound 1 ranging from 50 to 200 nM. The kinase reaction was initiated by the addition of ATP at concentrations ranging from 1 to 128 μM. Initial reaction velocities were plotted as a function of ATP concentration for each inhibitor concentration and fit to a competitive inhibition model. The double reciprocal plot showed a common y-intercept across inhibitor concentrations, consistent with competitive inhibition. From these data, the K_i_ of Compound 1 was determined to be 82 nM (95% confidence interval of 70–96 nM) (Fig. 2*E*).

### Co-crystal structure of Compound 1 bound to VRK1

Given the benzylidene-thiazolidinedione moiety in Compound 1, it was important to verify that it was binding VRK1 and was not merely a pan-assay interference compound affecting the TR-FRET assay readout. To confirm its binding mode with VRK1 and compare it with the predicted mode from computational docking, we determined the co-crystal structure of VRK1 with Compound 1 using macromolecular crystallography. Compound 1 emerged from the initial docking screen that included the conserved buried water molecule. In the docking pose, the phenolic hydroxyl group of Compound 1 was predicted to form a hydrogen bond with the backbone amide of Asp197 and to engage the conserved active-site water molecule, similar to previously published inhibitors (41, 42, 43). The true co-crystal structure closely recapitulated the predicted docking mode (Fig. 2*B*). Compound 1 was found in two out of the four VRK1 chains in the unit cell. In the co-crystal structure, the phenolic hydroxyl group participates in a hydrogen-bonding network involving the conserved active-site water molecule, the Lys71 side chain amino group, and the backbone amide of Asp197. One of the thiazolidinedione carbonyl groups of Compound 1 was shown to form a hydrogen bond with the backbone amide of the hinge residue, Phe134 (Fig. 2, *C* and *D*). Co-crystal structures of VRK1 with previously published inhibitors have demonstrated a hydrogen-bonding network between a difluorophenol moiety and water, Lys71, Glu83, Tyr87, and Asp197 (41, 42, 43). Here, the difluorophenol moiety is replaced with a dichlorophenol moiety, but the same interactions are observed.

### Kinetic parameters of VRK1-mediated phosphorylation of ULight-H3 are influenced by oligomerization

**Figure 3:**
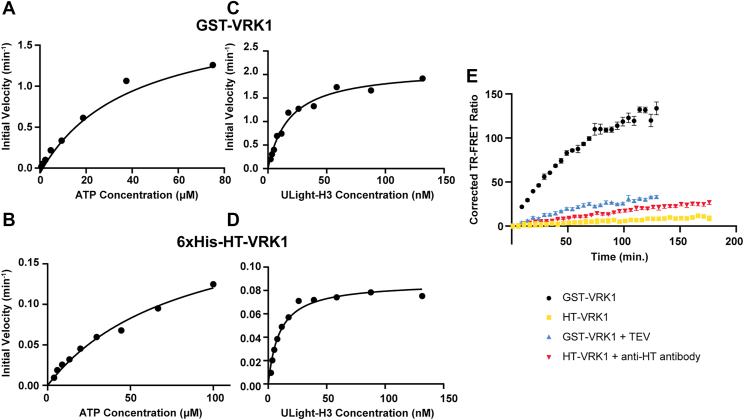
**V_max_, but not K_m_ of VRK1-mediated phosphorylation of ULight-H3, is impacted by VRK1 oligomerization.***A*, ATP titration against GST-VRK1 with constant ULight-H3. *B*, ATP titration against HT-VRK1 with constant ULight-H3. *C*, ULight-H3 titration against GST-VRK1 with constant ATP. *D*, ULight-H3 titration against HT-VRK1 with constant ATP. *E*, phosphorylation of ULight-H3 by VRK1 after cleavage of GST tag (GST-VRK1) and with induced dimerization of HT-VRK1. VRK1, vaccinia-related kinase 1.

Using the commercial TR-FRET activity assay, which measures phosphorylation of ULight-H3, we observed that GST-tagged VRK1 was substantially more active than VRK1 fused to monomeric tags, suggesting that dimerization may enhance catalytic activity. To test this, we determined the kinetic parameters of VRK1-mediated H3 phosphorylation. A previous study estimated the K_m,ATP_ of GST-VRK1 by titrating ATP from 500 μM to 15 nM, measuring ULight-H3 phosphorylation after a 30-min kinase reaction, and fitting the data to a one-site specific binding model to yield a K_m,__ATP_ of 2.8 μM (43). Although a reasonable approximation for estimating K_m,__ATP_, this assumes the reaction remains in the linear range throughout the 30-min time course, which is unlikely to hold at high ATP concentrations where substrate depletion and reaction plateauing may occur. To independently assess K_m,ATP_, we titrated ATP from 75 μM to 0.59 μM while keeping GST-VRK1 at a low concentration (10 nM) and ULight-H3 in excess (100 nM). H3 phosphorylation was measured over the course of 3 hours and initial velocities were determined from the early linear phase of each reaction. Fitting initial velocity *versus* ATP concentration to the Michaelis–Menten equation yielded a K_m,ATP_ of 38.8 μM (95% confidence interval of 26.1–60.3 μM) (Fig. 3*A*). Using a similar approach, we determined the K_m,app,H3_ to be 18.4 nM (95% confidence interval of 13.5–25.1 nM) by titrating ULight-H3 while keeping ATP and VRK1 constant (Fig. 3*C*). We next repeated the experiments using HaloTag-VRK1 instead of GST-VRK1. Because HaloTag, unlike GST, does not form dimers, we sought to determine whether artificial dimerization of VRK1 influences kinase activity. K_m,ATP_ and K_m,app,H3_ for HaloTag-VRK1 were found to be 82.4 μM (95% confidence interval of 52.0–143.9 μM) (Fig. 3*B*) and 9.6 nM (95% confidence interval of 7.3–12.6 nM) (Fig. 3*D*) respectively, values comparable to those of GST-VRK1. In contrast, V_max_ differed markedly: GST-VRK1 displayed V_max,ATP_ and V_max,app,H3_ values of 2.0 min^−1^ (95% confidence interval of 1.6–2.5 min^−1^) and 2.1 min^−1^ (95% confidence interval of 1.9–2.4 min^−1^) respectively, whereas HaloTag-VRK1 exhibited only 0.22 min^−1^ (95% confidence interval of 0.17–0.31 min^−1^) and 0.087 min^−1^ (95% confidence interval of 0.080–0.094 min^−1^). Thus, the monomeric and dimeric constructs of VRK1 bind both ATP and H3 with similar affinity, but catalysis is significantly faster in the dimeric construct.

To determine whether this effect reflects a construct-specific artifact or a general role for dimerization in VRK1 activation, we cleaved GST from GST-VRK1 using TEV protease. Compared with the GST-tagged construct, removal of GST reduced the initial velocity by 4.4-fold. Conversely, inducing dimerization of HaloTag-VRK1 using a bivalent HaloTag antibody increased the initial velocity by 2.6-fold (Fig. 3*E*). These findings suggest that artificial dimerization of VRK1 enhances activity in the TR-FRET assay.

### Tag-mediated dimerization does not affect VRK1 autophosphorylation

**Figure 4:**
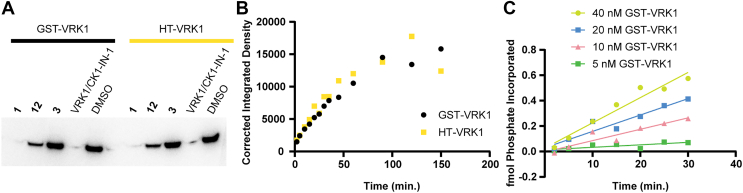
**Monomeric and GST-dimerized VRK1 behave similarly in radiometric assays.***A*, inhibition of VRK1 is independent of purification tag. *B*, VRK1 autophosphorylation rate is independent of purification tag. *C*, VRK1 autophosphorylation is slow and occurs primarily *in cis.* VRK, vaccinia-related kinase.

We were puzzled by the requirement for forced VRK1 dimerization to observe kinase activity in the TR-FRET assay and wanted to determine whether this requirement was universal across all substrates or specific to the ULight-H3 substrate. To address this, we performed [γ-^32^P]ATP autophosphorylation and transphosphorylation assays. Using [γ-^32^P]ATP, we measured the phosphorylation of various substrates including casein, stathmin 1, nucleosome, and myelin basic protein but found that even with excess exogenous substrate added, VRK1 preferred to phosphorylate itself. Therefore, we compared the autophosphorylation of VRK1 constructs bearing monomeric or dimeric tags. Tag-mediated dimerization of VRK1 did not affect VRK1 autophosphorylation or inhibition by an ATP-competitive inhibitor (Fig. 4, *A* and *B*).

The [γ-^32^P]ATP autophosphorylation assay is simple and highly sensitive, allowing rate constants to be measured in absolute units. We measured initial reaction velocities of autophosphorylation by quenching reactions at multiple time points and quantifying incorporation of ^32^P using a dot blot (Fig. 4*C*). When correcting for VRK1 concentration, the initial velocity curves overlapped, indicating that autophosphorylation likely occurs primarily *in cis*. We estimated the turnover rate of VRK1 in autophosphorylation to be ∼5.7 × 10^−6^ s^−1^. This extremely low rate is comparable to the inactive form of many other kinases (47, 48), suggesting VRK1 may be activated by other factors in cells.

### Design, synthesis, and characterization of fluorogenic active-site probes

We next developed a ligand displacement assay for VRK1, both to clarify observations from the TR-FRET ULight-H3 enzymatic assay and to create a low-cost screening method suitable for high-throughput use. We started with our top VRK1 inhibitor, Compound 1. Structural analysis indicated that its alkyl chain extended out of the active site into solvent and did not contribute significantly to binding (Fig. 2, *B*–*D*). Therefore, we hypothesized that we could exploit the lack of favorable binding interactions on this part of Compound 1 to append a fluorophore, with the goal of generating an ATP-competitive probe for binding displacement assays. Additionally, we opted to replace the dichlorophenol group of Compound 1 with the previously reported difluorophenol (41, 42, 43).

**Figure 5:**
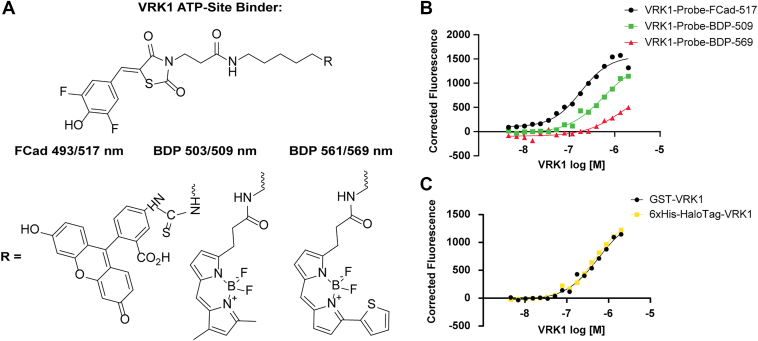
**Three fluorescent tool compounds that bind to the ATP site of VRK1.***A*, chemical structure of VRK1-binding scaffold and fluorophores appended to it. *B*, probe titration against VRK1 as read out by relief of fluorescence quenching: K_d_ values with 95% CI: VRK1-Probe-FCad-517: 180 nM (136–246 nM), VRK1-Probe-BDP-509: 575 nM (368–2031 nM), VRK1-Probe-BDP-569: no fit. *C*, VRK1-Probe-BDP-509 binding VRK1 is not dependent on purification tag. VRK, vaccinia-related kinase.

We evaluated probes with three different fluorophores selected for their spectral compatibility with terbium, enabling use in CoraFluor 1 donor TR-FRET assays. Probe structures and synthetic routes are reported in Figure 5*A* and Experimental Procedures. All three probes bound to VRK1 with comparable affinities, so the choice of fluorophore can be based on the assay format. All three probes were fluorogenic: they self-quenched in solution, but became fluorescent upon binding to VRK1. This property enabled quantification of binding using probe fluorescence as the readout. Using this assay, we determined dissociation constants of probes binding GST-VRK1 to be 180 nM (95% confidence interval of 136–246 nM) for VRK1-Probe-FCad-517 and 575 nM (95% confidence interval of 368–2031 nM) for VRK1-Probe-BDP-509. VRK1-Probe-BDP-569 was less fluorogenic than the other probes and thus we could not calculate an accurate K_d_ for this probe using the fluorogenic binding assay (Fig. 5*B*). Unlike the TR-FRET activity assay, in which VRK1 activity differed depending on monomeric or dimeric tag, probe K_d_ values were unaffected by the purification tag (Fig. 5*C*). Thus, probe-based displacement assays provide a method for quantifying ATP-site inhibitors without relying on artificial dimerization of the protein.

### Validation of the probe displacement assay

**Figure 6:**
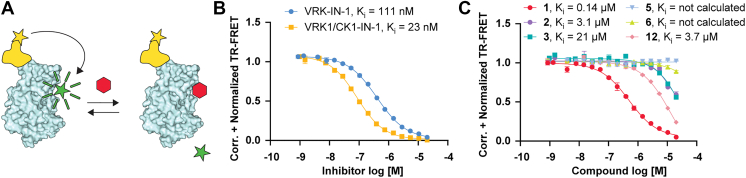
**Compound 1–based fluorescent probes can be used to screen VRK1 ATP-site inhibitors.***A*, schematic of TR-FRET–based probe displacement assay. *B*, dose-response curves for two published inhibitors. *C*, dose-response curves for TR-FRET activity assay hits. Indicated K_i_ values were obtained by applying the Cheng-Prusoff equation to IC_50_ values determined by fitting data to the four-parameter sigmoidal dose-response equation. VRK, vaccinia-related kinase; TR-FRET, time-resolved fluorescence resonance energy transfer.

To measure ligand displacement, we implemented a TR-FRET assay rather than relying on the fluorogenic readout because several probes did not exhibit sufficient fluorescence enhancement to accurately determine K_d_ values. The time-resolved, gated detection of TR-FRET also affords higher sensitivity than simple fluorescence intensity measurements. We evaluated both previously published inhibitors and our novel scaffolds using a CoraFluor 1–conjugated nanobody against the VRK1 purification tag (either GST or HaloTag) as the FRET donor and VRK1-Probe-BDP-569 as the FRET acceptor (Fig. 6*A*). We titrated inhibitors against a constant concentration of fluorescent probe and CoraFluor 1–labeled GST-VRK1, calculated the IC_50_ values, and then used the Cheng-Prusoff equation to convert IC_50_ values to K_i_ values. The leading VRK1 inhibitor, VRK1/CK1-IN-1, with a published K_i_ of 37.9 nM as determined by the commercial kinase activity assay (42), was measured to have a K_i_ of 23 nM in our probe displacement assay (Fig. 6*B*). K_i_ values for additional compounds, as measured by kinase activity and by probe displacement TR-FRET, are found in Figure 6, *B* and *C*, and Table S2. Overall, the strong agreement between published K_i_ values and those obtained using the probe displacement assay validates the performance of the new probes and assay format.

## Discussion

VRK1 is of increasing interest as a drug target in glioblastoma and other VRK2-low cancers. Here, we used computational docking to screen a large library of diverse chemotypes. From this screen, we identified a potent VRK1 inhibitor (K_i_ = 82 nM) that is selective for VRK1 over VRK2. While Compound 1 shares the dihalophenol ring moiety with both VRK-IN-1 and VRK1/CK1-IN-1, Compound 1 and VRK1/CK1-IN-1 are selective for VRK1 over VRK2, while VRK-IN-1 is a pan-VRK inhibitor. Further SAR on compounds that share the dihalophenol ring moiety but have different core scaffolds that confer selectivity between VRK1 and VRK2 will be a promising starting point for future drug discovery efforts.

Drug discovery benefits from multiple assays. We employed three different biochemical assays in this work, including our novel probe displacement assay, and summarize their strengths and weaknesses in Table 1. The commercial TR-FRET assay using ULight-H3 peptide has the advantages of measuring catalytic activity and not requiring radioactivity, but it requires artificial dimerization of the kinase for unclear reasons. Previous groups have shown that GST-VRK2, but not His-VRK2, is active in the commercial TR-FRET assay (42, 43). Our results provide a rationale for this construct dependence and underscore the need to account for it when using the commercial kinase assay.

**Table 1:** Characterization of three assays useful in VRK1 biochemistry and inhibitor development

Assay	Pros	Cons
ULight-H3 Phosphorylation TR-FRET	• High-throughput • Useful for screening and comparing inhibitors • Agnostic towards mode of inhibition • Can compare VRK1 to VRK2	• Run at the fringes of steady-state • Dependent on dimeric purification tag • Expensive
Radiometric autophosphorylation	• Simple – primary readout • Useful to obtain biochemical parameters • Can compare VRK1 to VRK2 • Tag-agnostic	• Low-throughput • Difficult to measure other substrates as autophosphorylation dominates
Fluorescent Probe Binding	• Simple – one-pot binding assay • Ability to run as fluorescence quenching release without additional fluorophores and in TR-FRET • Useful for screening and comparing ATP-site inhibitors • Inexpensive • Tag-agnostic	• Cannot screen for non-ATP–competitive inhibitors • Not useful for VRK2

Artificial dimerization is not required for the phosphorylation of casein, stathmin 1, myelin basic protein, or autophosphorylation as measured by [γ-^32^P]ATP incorporation. We suspect that histone H3 peptide alone may be a poor substrate of VRK1. Recent work suggested that VRK1 phosphorylates histone H3 through interaction with nucleosome acidic patches and is inactive against H3 alone (5). We suspect that the ULight fluorophore in the commercial kit may function as a weak VRK1 active site inhibitor, thus positioning H3 for the other VRK1 monomer in the dimer to phosphorylate it. Addition of ULight-H3, but not FITC-H3, to the radiometric autophosphorylation assay inhibited autophosphorylation. Furthermore, FITC-H3 was inactive in the TR-FRET activity assay, suggesting that the ULight fluorophore influences activity. Further work is needed to determine whether the ULight fluorophore functions as a weak VRK1 inhibitor and to determine if GST-VRK1 exhibits increased activity toward free histone H3 and nucleosomal H3.

We report the first active-site probe displacement assays for VRK1. Our probes are not sensitive to the dimerization state of VRK1 and allow for inexpensive and rapid screening and quantification of ATP-site VRK1 inhibitors. These probes can be used to screen inhibitors in a TR-FRET assay measuring energy transfer from a lanthanide donor fluorophore conjugated to the VRK1 purification tag to the probe. The long fluorescence lifetime of the lanthanide allows for the time delay between reading donor and acceptor emission and limits the background fluorescence. However, not all labs may have access to the plate readers required to measure a TR-FRET assay. Thus, the inhibitors can also be screened by a simple plate reader single-wavelength fluorescence assay in which probe displacement from the active site results in decreased fluorescence due to the fluorogenic nature of the probes.

An enduring puzzle is the remarkably low catalytic activity of purified VRK1. We determined the VRK1 autophosphorylation rate to be approximately 5.7 × 10^−6^ s^−1^, comparable to rates observed for inactive forms of many other kinases. In the commercial TR-FRET assay and in radiometric phosphorylation assays, VRK1-mediated phosphorylation is within the linear range for over an hour under steady-state conditions, suggesting very low k_cat_ in this assay as well. Absolute k_cat_ values were not reported in other papers, but reading the methods used suggests that very low k_cat_ values are common to all biochemical studies with pure VRK1. It seems likely that VRK1 exhibits higher catalytic activity in cells. VRK1 is not known to be regulated by activation loop phosphorylation. It may instead be activated by binding partners such as nucleosomes or by posttranslational modification. Much remains to be learned concerning the biochemistry and biology of this interesting kinase, which is increasingly recognized as a promising drug target.

## Experimental procedures

### *In silico* library generation

The entire MolPort library of approximately eight million compounds (as of August 2021) was filtered down to four million by removing compounds meeting the following criteria: duplicates, heavy atom count > 20, pan-assay interference compound, undesirable functionality, hydrogen bond donors > 4, hydrogen bond acceptors > 8, undefined chiral centers > 1, logP > 5, rotatable bonds > 10. We filtered for compounds with topological surface area between 50 and 150 Å^2^ and molecular weight between 300 and 500 Da. In addition, the top 10,000 most diverse compounds with a drug-likeness score < 5 as computed in DataWarrior were removed to further prune highly functionalized molecules.

### Glide docking

The crystal structure of VRK1 co-crystallized with a bis-difluorophenol-aminopyridine inhibitor was obtained from the PDB (6BU6) (43). Using Epik within Schrӧdinger Maestro, the protein was prepared to assign bond orders, add hydrogens, create disulfide bonds, and generate optimal protonation and tautomer states. In one protein preparation, all water molecules were deleted. In a separate protein preparation, all waters except those within 5 Å of the ligand were deleted. Prime was used to complete missing side chains. A restrained minimization of the protein structure was performed using the default constraint of 0.30 Å RMSD and the OPLS4 force field. The binding site was defined around the co-crystallized ligand and all other defaults were used. Molecular docking simulations were first performed using Glide HTVS (high-throughput virtual screening). The top approximately 50,000 compounds with the lowest docking scores were then docked again using Glide SP (standard precision). Docked compounds were then filtered for the formation of a hydrogen bond with the kinase hinge residue, Phe134, with maximum hydrogen bond distance set to 2.8 Å, minimum hydrogen bond donor angle of 120°, and minimum hydrogen bond acceptor angle of 90°. Compounds that were docked with the protein prepared with water molecules were additionally filtered for the formation of a hydrogen bond with the conserved buried water molecule. Compounds were further filtered using Strain Energy Calculation and Rescoring using the 4RDDD solvent model, and compounds with strain energy > 4.0 kcal/mol were removed from downstream analysis. Using Structural Interaction Fingerprints, compounds were clustered based on interactions with polar residues, hydrogen bond acceptors, hydrogen bond donors, and charged residues. The compounds with the best (most negative) docking score in each cluster were then evaluated *via* graphical analysis of docked poses to select for those with favorable interactions with the protein, including multiple hydrogen bonds and reasonable hydrogen bonding angles, as well as overall solvation properties.

### Protein expression and purification

Full-length VRK1 (pDONR223-VRK1, Addgene # 23496) was cloned into pGEX2T-CHMP4A (Addgene # 80632) using Gibson assembly. GST-VRK1 was expressed in Rosetta 2 (DE3) competent cells (Novagen). Cells were induced with 1 mM IPTG (Research Products International Corporation) at an optical density of 0.6 at 600 nm and grown for 16 to 18 h at 18 °C. Cells were pelleted by centrifugation and resuspended in 50 mM Tris, pH 7.5, 150 mM NaCl, 0.05% NP-40, 5% glycerol, 3 mM DTT, 1 mM PMSF (Sigma Life Sciences), 0.25 mg/ml lysozyme (Sigma Life Sciences), and cells were lysed by sonication. The lysate was then clarified by ultracentrifugation and the soluble fraction was subjected to glutathione affinity purification using Pierce Glutathione Agarose. Bound protein was eluted in 50 mM Tris, pH 7.5, 150 mM NaCl, 5% glycerol, 3 mM DTT, and 20 mM glutathione and subsequently buffer-exchanged into 20 mM sodium phosphate, pH 7.2, 3 mM DTT, 5% glycerol with dialysis using Spectrum Laboratories Spectra/Por MolecularPorous membrane tubing with molecular weight cutoff of 6 to 8 kDa for 16 to 18 h. Protein was then concentrated using Amicon Ultra centrifugal filters with a molecular weight cutoff of 50 kDa and further purified with cation exchange chromatography using a HiPrep SP FF column (Cytiva Life Sciences). Bound protein was eluted with a linear gradient from 0 M NaCl to 1.5 M NaCl in 20 mM sodium phosphate, pH 7.2, 5% glycerol, 3 mM DTT. Protein was then concentrated and buffer-exchanged into 100 mM potassium glutamate, pH 7.7, 50 mM HEPES, 1 mM DTT, 10% glycerol, then flash frozen in liquid nitrogen and stored at −80 °C. Protein purity was determined by SDS-PAGE and protein was quantified using A_280_ and the extinction coefficient 107,150 M^−1^ cm^−1^ and separately using bicinchoninic acid assay.

His_6_-HaloTag-VRK1 was purchased from Twist Bioscience and transformed into Rosetta 2 (DE3) competent cells. Cells were induced with 1 mM IPTG at an optical density of 0.6 at 600 nm and grown for 16 to 18 h at 18 °C. Cells were pelleted by centrifugation and resuspended in 20 mM Tris, pH 7.5, 500 mM NaCl, 3 mM DTT, 5% glycerol, 1 mM PMSF, and 0.25 mg/ml lysozyme, and cells were lysed by sonication. The lysate was then clarified by ultracentrifugation and the soluble fraction was subjected to HisPur-Ni-NTA resin (Thermo Fisher Scientific). Bound protein was eluted in 20 mM Tris, pH 7.5, 500 mM NaCl, 400 mM imidazole, 3 mM DTT, and 5% glycerol and subsequently buffer-exchanged into 20 mM Tris, pH 7.5, 500 mM NaCl, 3 mM DTT, 5% glycerol using Spectrum Laboratories Spectra/Por MolecularPorous membrane tubing with molecular weight cutoff of 6 to 8 kDa for 16 to 18 h. Protein was concentrated using Amicon Ultra centrifugal filters with a molecular weight cutoff of 50 kDa. Concentrated protein was flash frozen in liquid nitrogen and stored at −80 °C. To remove free HaloTag impurity, protein was thawed and buffer-exchanged into 20 mM Tris, pH 7.7, 20 mM NaCl, 5% glycerol, and 3 mM DTT. Protein was loaded onto Mono Q 4.6/100 PE column *via* Cytiva ÄKTA pure FPLC system. Protein was eluted using a gradient from 20 mM Tris, pH 7.7, 20 mM NaCl, 5% glycerol, and 3 mM DTT to 20 mM Tris, pH 7.7, 1 M NaCl, 5% glycerol, and 3 mM DTT. Eluted fractions were verified by SDS-PAGE and fractions containing HT-VRK1 with limited free HaloTag were pooled, concentrated, and buffer-exchanged into storage buffer (20 mM Tris, pH 7.7, 20 mM NaCl, 10% glycerol, 3 mM DTT), flash frozen, and stored at −80 °C.

His_6_-GST-VRK2 was purchased from Twist Bioscience and transformed into Rosetta 2 (DE3) competent cells. Cells were induced with 1 mM IPTG at an optical density of 0.6 at 600 nm and grown for 16 to 18 h at 18 °C. Cells were pelleted by centrifugation and resuspended in 20 mM Tris, pH 7.5, 500 mM NaCl, 3 mM DTT, 5% glycerol, 1 mM PMSF, and 0.25 mg/ml lysozyme, and cells were lysed by sonication. The lysate was then clarified by ultracentrifugation and the soluble fraction was subjected to HisPur Ni-NTA resin. Bound protein was eluted in 20 mM Tris, pH 7.5, 500 mM NaCl, 400 mM imidazole, 3 mM DTT, 5% glycerol, and subsequently buffer exchanged into 20 mM Tris, pH 7.5, 500 mM NaCl, 3 mM DTT, 5% glycerol with dialysis using Spectrum Laboratories Spectra/Por MolecularPorous membrane tubing with molecular weight cutoff of 6 to 8 kDa for 16 to 18 h. Protein was then subjected to glutathione affinity purification utilizing Pierce Glutathione Agarose. Bound protein was eluted in 20 mM Tris, pH 7.5, 500 mM NaCl, 3 mM DTT, 5% glycerol, 20 mM glutathione, and buffer-exchanged into 20 mM Tris, pH 7.5, 500 mM NaCl, 3 mM DTT, 10% glycerol using Amicon Ultra centrifugal filters with molecular weight cutoff of 50 kDa. Concentrated protein purity was determined by SDS-PAGE and quantified using A_280_ with an extinction coefficient of 97,180 M^−1^ cm^−1^.

### Co-crystallization of VRK1 with Compound 1

A truncated human VRK1 construct (residues 3–364) previously established for crystallization was used (41, 42, 43). This construct includes four clusters of surface-entropy reduction mutations in which solvent-exposed Lys/Glu residues were mutated to alanine (K34A/K35A/E36A, E212A/ K214A/E215A, E292A/K293A/K295A, and K359A/K360A), as well as M1A and M2P mutations. The gene was fused to an N-terminal His_6_ tag with a TEV protease cleavage site, cloned into pNIC28-Bsa4, and expressed in *Escherichia coli* BL21(DE3)-R3 cells co-expressing λ-phosphatase. Terrific Broth (50 μg/ml kanamycin, 35 μg/ml chloramphenicol) was used to grow cultures at 37 °C to OD_600_ ∼ 3, cooled to 18 °C, and induced with 0.1 mM IPTG overnight. Cells were collected by centrifugation, resuspended in buffer (50 mM HEPES, pH 7.5, 500 mM NaCl, 10 mM imidazole, 0.5 mM TCEP, protease inhibitors), and lysed by sonication. Clarified lysates were purified on Ni–Sepharose resin, eluted with 300 mM imidazole, and incubated with TEV protease overnight at 4 °C while dialyzing against gel filtration buffer (20 mM HEPES, pH 7.5, 300 mM NaCl, 0.25 mM TCEP, 5% glycerol). VRK1 was further purified following reverse Ni-affinity by size-exclusion chromatography (Superdex 200 16/60). Lyophilized Compound 1 was dissolved in VRK1 solution at a sixfold molar excess, preincubated on ice, and concentrated to 6 mg/ml as measured by A_280_ on a Nanodrop spectrophotometer. Crystallization trials were set up using previously successful precipitant solutions available at the PDB. Well-diffracting crystals were obtained under the following precipitant condition: 30.25% PEG 3350, 0.2 M Li_2_SO_4_, 0.1 M SBG (sodium tartrate dihydrate, bis-tris, glycylglycine), pH 6.5. Crystallization drops were set up at a 1:2 protein:reservoir ratio at 20 °C. Crystals were cryoprotected with 25% glycerol and flash-cooled in liquid nitrogen. Diffraction data were collected at MANACÁ beamline at CNPEM-Sirius-LNLS synchrotron source and processed with XDS (49). Because the crystals were isomorphous to PDB entry 6AC9 (50), this structure was used directly as a starting model for refinement in PHENIX.refine (51), and model building was carried out in Coot (52). Validation was performed with MolProbity (53). The VRK1 residues 20 to 342 were modeled for chain A, 21 to 43 and 49 to 341 for chain B, 22 to 43 and 49 to 341 for chains C and D. The following side chains were omitted owing to insufficient electron density: chain A: F48, E60, K104, K106, K124, K140, K188, K218, K266, N294, K301, and K329; chain B: E60, E94, K104, K106, K124, Y187, K218, C220, H221, K301, and K334; chain C: Q22, F23, I28, K38, L41, I43, C50, M56, E60, D76, N77, K121, N122, K124, K140, K188, K218, K266, K269, N294, and K338; and chain D: Q22, L41, S59, E60, N77, K106, K121, N122, K124, K140, K188, and K269. Additional electron density nearby protein atoms was modeled as two molecules of the inhibitor Compound 1, 13 ions, five glycerol molecules, four buffer molecules, and nine PEG molecules. Our structure is similar to other VRK1 tetrameric structures and the final model presents R/Rfree of 18.1/21.6 and a MolProbity score of 1.08. Compound 1 coordinates and structure factors were deposited in the PDB (code 9ZKG; Table S3).

### Nanobody labeling

Anti-rabbit IgG antibody (CTK0101 ChromoTek cat. # shurbGNHS-1), anti-GST VHH (Abcam cat. # ab19256), and anti-HaloTag VHH (ChromoTek cat. # OT-250) were labeled as previously described (54).

### TR-FRET kinase activity high-throughput screen and inhibitor dose-response

Compounds selected from the docking screen were tested *in vitro* using Revvity’s TR-FRET kinase activity assay in 20 μl reactions in white 384-well plates (Greiner Bio-One cat. # 784075). Briefly, GST-VRK1 was preincubated at room temperature with 10 or 30 μM compound for 30 min in kinase buffer (50 mM HEPES, pH 7.5, 5 mM MgCl_2_, 1 mM EGTA, 2 mM DTT, 0.01% Tween-20). Following compound preincubation, ULight-histone H3 peptide (Revvity cat. # TRF0125) and ATP (Promega cat. # V915A) were added and the reaction was incubated at room temperature for 75 min. Kinase reaction final concentrations were 14 nM GST-VRK1, 50 nM ULight-H3, and 2.8 μM ATP. Kinase reactions were quenched with 8 mM EDTA and europium anti-phosphohistone H3 (Thr3) antibody was added to a final concentration of 2 nM and incubated at room temperature for 1 h prior to reading on an EnVision plate reader (Revvity) with 302/75 nm excitation and 665/75 nm (ULight) and 615/8 nm (europium) emissions. Compound inhibition was normalized to the DMSO vehicle control.

Compounds with >∼10% inhibition of VRK1 at >10 μM were then titrated from 100 μM down to 0.1 nM under the same assay conditions as described above. Dose-response data were fit to a sigmoidal four-parameter logistic (4 PL) model in GraphPad Prism to determine IC_50_ values. Compounds with IC_50_ values <∼30 μM were then tested against GST-VRK2 under the same assay conditions as described above.

### TR-FRET kinase activity assay to measure K_m_ and V_max_

To establish K_m,ATP_ for GST-VRK1, ATP was titrated from 300 μM to 1.18 μM (twofold serial dilution, 2X final concentration) in kinase buffer. Ten microliters of each 2X ATP concentration was added in duplicate to wells on a white 384-well plate, and 10 μl of kinase buffer without ATP was added in duplicate as a negative control. To each well, 10 μl of 20 nM GST-VRK1 (2X final concentration), 200 nM ULight-H3 (2X final), 2.5 nM anti-phospho-H3 (Thr3) rabbit IgG antibody *(Cell Signaling cat. # 13576)* (2X final), and 5 nM CoraFluor 1–labeled anti-IgG nanobody *(ChromoTek cat. # shurbGNHS-1)* (2X final) in kinase buffer was added and TR-FRET measurements were immediately acquired on an EnVision plate reader (Revvity) with 320/75 nm excitation and 665/75 nm (ULight) and 615/8 nm (terbium) emissions and subsequently read every 10 min for 3 h. The TR-FRET ratio was taken as the 665/615 nm intensity ratio. The assay floor (background) was defined as the average of the TR-FRET ratio of the wells lacking ATP for each time point. Data were background-corrected by subtracting out the background TR-FRET ratio from each ATP-containing well at each time point. The background-corrected TR-FRET ratios for each ATP concentration were plotted over time to determine initial velocities. Initial velocity was unable to be determined for the 150 μM ATP concentration because the reaction was too fast to accurately measure the linear range. Thus, 150 μM ATP was left out of the final Michaelis–Menten fit. Initial velocities were then plotted against ATP concentration and fit to a Michaelis–Menten model in GraphPad Prism.

To establish K_m,ATP_ for HaloTag-VRK1, ATP was titrated from 200 μM to 0.68 μM (1.5-fold serial dilution, 2X final concentration) in kinase buffer. 7.5 μl of each ATP concentration was added in duplicate to wells on a white 384-well plate, and 7.5 μl of kinase buffer without ATP was added in duplicate as a negative control. To each well, 7.5 μl of 8 nM HT-VRK1 (2X final concentration), 200 nM ULight-H3 (2X final), 5 nM anti-phospho-H3 (Thr3) rabbit IgG antibody (2X final), and 10 nM CoraFluor 1-labeled anti-IgG nanobody (2X final) in kinase buffer was added and TR-FRET measurements were immediately acquired on an EnVision plate reader as described above. Initial velocities were determined as described above. Initial velocity traces for ATP concentrations <3.9 μM could not be reliably fit because activity was too low. Thus, the final Michaelis–Menten fit did not include ATP concentrations <3.9 μM.

To establish K_m,app,ULight-H3_ for GST-VRK1 and HT-VRK1, ULight-H3 was titrated from 264 nM to 4.6 nM (1.5-fold serial dilution, prepared as 2X stocks) in kinase buffer. Because the low end of ULight-H3 concentrations were less than the kinase concentration, K_m,app,ULight-H3_ and V_max,app,ULight-H3_ are reported rather than absolute values. 7.5 μl of each ULight-H3 concentration was added in quadruplicate to wells on a white 384-well plate. To two out of the four wells for each ULight-H3 concentration, 7.5 μl of 8 nM of the corresponding VRK1 (2X final concentration), 100 μM ATP (2X final), 5 nM anti-phospho-H3 (Thr3) antibody (2X final), and 10 nM CoraFluor 1-labeled anti-IgG nanobody (2X final) in kinase buffer was added. To the additional two wells for each ULight-H3 concentration, 7.5 μl of 100 μM ATP (2X final), 5 nM anti-phospho-H3 (Thr3) antibody (2X final), and 10 nM CoraFluor 1-labeled anti-IgG nanobody (2X final) in kinase buffer was added. TR-FRET measurements were immediately acquired on an EnVision plate reader as described above and subsequently read every 5 min for 3 h. The background for each ULight-H3 concentration was defined as the average TR-FRET ratio of wells lacking VRK1 at that ULight-H3 concentration and time point. Data were background-corrected by subtracting out the background TR-FRET ratio for each ULight-H3 concentration and time point from the corresponding VRK1-containing wells. The background-corrected TR-FRET ratio for each ULight-H3 concentration was plotted over time to determine initial velocities. Initial velocities were then plotted against ULight-H3 concentration and fit to a Michaelis–Menten model in GraphPad Prism.

### TR-FRET kinase activity assay to measure Compound 1 K_i_ values

To determine the K_i_ value for Compound 1 against VRK1, 7.5 μl of 8 nM GST-VRK1 (2X final), 150 nM ULight-H3 (2X final), 5 nM anti-phospho-H3 (Thr3) antibody (2X final), and 10 nM CoraFluor 1–labeled anti-IgG nanobody (2X final) in kinase buffer was added to the wells of a white 384-well plate. Using an HP D300e drug dispenser, each concentration of Compound 1 (0, 50, 100, 200 nM in 100% DMSO) was added across eight VRK1-containing wells on the plate. All wells, including Compound 1–lacking wells, were normalized to the same final DMSO concentration. VRK1 with Compound 1 (or DMSO control) was incubated at room temperature for 26 min. ATP was serially diluted in kinase buffer from 256 μM to 2 μM (8-point dilution series as 2X final concentration, plus kinase buffer only control). To each pair of wells at a given Compound 1 concentration, 7.5 μl of an ATP solution was added, such that each ATP concentration was tested in duplicate at each Compound 1 concentration. TR-FRET measurements were immediately acquired on an EnVision plate reader with 320/75 nm excitation and 665/75 nm (ULight) and 615/8 nm (terbium) emissions and subsequently read every 4 min for 3 h. The TR-FRET ratio was taken as the 665/615 nm intensity ratio. The background was defined as the average TR-FRET ratio of wells lacking both Compound 1 and ATP at each time point. Data were background-corrected by subtracting the background TR-FRET ratio from each ATP-containing well and subsequently normalized by dividing by DMSO control signal at each time point. The background-corrected and normalized TR-FRET ratio for each ATP concentration and Compound 1 concentration was plotted over time to determine initial velocities. Initial velocities were then plotted against ATP concentration at each Compound 1 concentration and fitted to a competitive inhibition model in GraphPad Prism.

### TR-FRET kinase activity assay with dimerization tuning via TEV and anti-HaloTag

GST-VRK1 was incubated with 0X, 5X, 10X, and 50X TEV protease (GenScript cat. #Z03030) for 5 h at room temperature. To a 384-well plate, 10 nM GST-VRK1 treated with TEV or kinase buffer control, 100 nM ULight-H3, 50 μM ATP, 2.5 nM anti-phospho-H3 (Thr3) antibody, and 5 nM CoraFluor 1–labeled anti-IgG nanobody in kinase buffer was added and TR-FRET measurements as previously described were acquired every 5 min for 2 h. Data were background-corrected by subtracting the TR-FRET ratio of wells containing all components except VRK1 at each time point.

HaloTag-VRK1 was briefly preincubated with anti-HaloTag antibody (Promega cat. # G9211) at room temperature. To a 384-well plate, 10 nM HT-VRK1 treated with anti-HaloTag or kinase buffer control, 100 nM ULight-H3, 50 μM ATP, 2.5 nM anti-phospho-H3 (Thr3) antibody, and 5 nM CoraFluor 1-labeled anti-IgG nanobody in kinase buffer were added and TR-FRET measurements as previously described were acquired every 5 min for 2.5 h. Data were background-corrected by subtracting out the background TR-FRET ratio (all components except VRK1) from each time point.

### Inhibition of VRK1 autophosphorylation as measured by [γ-^32^P]VRK1 band on SDS-PAGE

GST-VRK1 or HaloTag-VRK1 were preincubated with compound for 30 min at room temperature. To VRK1 and compound solutions, hot and cold ATP were added to final concentrations of 100 nM VRK1, 5 μM cold ATP, 0.05 μCi/μl [γ-^32^P]ATP (Revvity cat. # BLU502A), and 20 μM compound. Reactions were run for 25 min and then quenched with 15 mM EDTA, 1X NuPAGE LDS sample buffer (Thermo Fisher Scientific cat. # NP0007), and 5% 2-mercaptoethanol. Samples were boiled for 5 min at 95 °C and loaded on a NuPAGE Bis-Tris protein gel (Invitrogen cat. # NP0336). The gel was run at approximately 180 V for 40 min. Gel was stained with InstantBlue Coomassie Protein Stain (Abcam cat. # ab119211) and imaged for total protein visualization. Gel was then dried and exposed on a blanked phosphor screen for 16 to 18 h prior to imaging with a Cytiva Amersham Typhoon phosphorimager.

### Radiometric dot blot comparison of GST-VRK1 and HT-VRK1 autophosphorylation activity

GST-VRK1 or HT-VRK1 was added to a solution of hot and cold ATP to the following final concentrations: 10 nM VRK1, 500 nM cold ATP, 0.02 μCi/μl hot ATP in kinase buffer. The reaction was quenched by removing 5 μl of reaction and adding it to 5 μl of quench solution (25 mM EDTA, 10 mM cold ATP in kinase buffer) at the following time points: 2, 5, 10, 15, 20, 25, 30, 35, 45, 60, 90, 120, 150 min. Three microliters of each quenched reaction was spotted on nitrocellulose paper and allowed to air dry in a laminar flow hood. The filter paper was subsequently washed 3 × 10 min with 1 M NaCl and 100 mM cold ATP and then once with water before being allowed to fully air dry in a laminar flow hood. The dried nitrocellulose paper was exposed for 16 to 18 h on a blanked phosphor screen prior to imaging with a Cytiva Amersham Typhoon phosphorimager. Dots were quantified in ImageJ by subtracting background using a rolling ball radius of 25 pixels and then quantifying the integrated density of each spot using a fixed radius circle to surround each spot. Integrated density was plotted against time in GraphPad Prism.

### Determination of turnover rate of GST-VRK1 autophosphorylation

GST-VRK1 was added to a solution of hot and cold ATP to the following final concentrations in kinase buffer: 5, 10, 20, or 40 nM VRK1, 500 nM cold ATP, 0.02 μCi/μl hot ATP. A 0 min time point was obtained by adding the same final concentrations of cold and hot ATP to kinase solutions that had been pre-quenched. The reaction was quenched by removing 5 μl of the reaction and adding it to 5 μl of quench solution at the following time points: 2, 5, 10, 15, 20, 25, 30 min. Four microliters of each quenched reaction was spotted on nitrocellulose paper and allowed to air-dry. The filter paper was subsequently washed 3 × 10 min with 1 M NaCl and 100 mM cold ATP, followed by a single wash with water before being allowed to fully air dry in a laminar flow hood. Separately, 1 μl of the initial hot and cold ATP solution was diluted 1000-fold in kinase buffer and subsequently serially diluted twofold for eight total concentrations. Four microliters of each ATP solution was spotted on the same nitrocellulose membrane as the kinase reaction spots and the filter paper was allowed to dry. The dried nitrocellulose paper was exposed for 16 to 18 h on a blanked phosphor screen prior to imaging with a Cytiva Amersham Typhoon phosphorimager. Dots were quantified in ImageJ using a fixed-radius circular region of interest and calculating the integrated density of each dot. Data were background-corrected by subtracting the integrated density of the 0 min dot for the corresponding VRK1 concentration at each time point. The ATP-only dots were used to generate a standard curve in which integrated density was plotted against amount of ATP in the dot. The standard curve yielded the following equation:

intden=8434x+2047

where the slope is in units IntDen/fmol phosphate and the y-intercept is in units IntDen. The slope and y-intercept of the standard curve were used to translate integrated densities to fmol phosphate for each reaction point. Background-corrected fmol phosphate was then plotted against time (min) for each VRK1 concentration in GraphPad Prism.

### Fluorogenic probe binding

GST-VRK1 or HaloTag-VRK1 was titrated from 1333 nM down to 4.6 nM (1.5-fold serial dilution) in kinase buffer in a black 384-well plate. Using an HP D300e drug dispenser, VRK1-Probe-FCad-517, VRK1-Probe-BDP-509, and VRK1-Probe-BDP-569 were added to each well at 20 nM. Fluorescence was read out using an Agilent BioTek plate reader (green probes: excitation 485 nm/emission 535 nm; red probe: excitation 543 nm/emission 571 nm). Background subtraction was performed by subtracting the fluorescence of each probe in kinase buffer without VRK1 from the corresponding wells. Fluorescence was plotted against VRK1 concentration in GraphPad Prism and data were fit to a sigmoidal four-parameter logistic (4 PL) curve.

### TR-FRET compound displacement

To determine K_d,app_ values for VRK1-Probe-BDP-569, 15 μl of 5 nM GST-VRK1, 5 nM CoraFluor 1–labeled GST VHH nanobody in kinase buffer was added to wells on a white 384-well plate. 5 nM CoraFluor 1–labeled GST nanobody without VRK1 was added to additional wells as a control. Using an HP D300e drug dispenser, VRK1-Probe-BDP-569 in 100% DMSO was dispensed from 1 μM to 1 nM (16-point dilution series) in both VRK1-containing wells and wells lacking VRK1. VRK1 was incubated with VRK1-Probe-BDP-569 for approximately 30 min before TR-FRET measurements were acquired using an EnVision plate reader: 337 nm laser excitation, 570/8 nm (BODIPY red) emission, 485/14 nm (terbium) emission. The TR-FRET ratio was taken as the 570/485 nm intensity ratio. The background for each VRK1-Probe-BDP-569 concentration was defined as the average TR-FRET ratio of wells lacking VRK1 at that probe concentration. Data were background-corrected by subtracting this background TR-FRET ratio from the corresponding VRK1-containing wells. Background-corrected TR-FRET ratio was plotted against VRK1-Probe-BDP-569 concentration, and data were fit to a sigmoidal 4 PL model in GraphPad Prism to determine K_d,app_ values.

To determine the K_i_ values for selected inhibitors, 15 μl of 5 nM GST-VRK1, 5 nM CoraFluor 1–labeled GST VHH nanobody in kinase buffer was added to wells on a 384-well plate. 5 nM CoraFluor 1–labeled GST nanobody without VRK1 was added to additional wells as a control. Using an HP D300e drug dispenser, 100 nM of VRK1-Probe-BDP-569 was added to each well, and selected inhibitors were titrated from 20 μM to 1 nM (16-point dilution series). All wells, including inhibitor-lacking wells, were normalized to the same final DMSO concentration. The plate was incubated at room temperature for approximately 30 min and then TR-FRET measurements were acquired using an EnVision plate reader with 337 nm laser excitation, 570/8 nm (BODIPY red), and 485/14 nm (terbium) emissions. The TR-FRET ratio was taken as the 570/485 nm intensity ratio. The background was defined as the average TR-FRET ratio of wells lacking VRK1. Data were background-corrected by subtracting this background TR-FRET ratio and subsequently normalized by dividing by the DMSO control. Background-corrected and normalized TR-FRET ratio was plotted against inhibitor concentration, and data were fit to a sigmoidal four-parameter logistic (4 PL) model in GraphPad Prism to determine IC_50_ values. IC_50_ values were then converted to K_i_ values using VRK1-Probe-BDP-569 concentration in the Cheng-Prusoff equation.

### Chemistry

All chemicals and solvents of analytical grade were obtained from commercial sources and used without further purification.

### Synthesis of (Z)-3-(5-(3,5-difluoro-4-hydroxybenzylidene)-2,4-dioxothiazolidin-3-yl)propanoic acid

3-(2,4-dioxo-thiazolidin-3-yl)-propionic acid (1 eq), 3,5-difluoro-4-hydroxybenzaldehyde (1 eq), and piperidine (3 eq) in methanol were added to a 2-neck round-bottom flask equipped with a stir bar in a mineral oil bath at room temperature for 30 min. At 30 min, the mineral oil bath was heated to 70 °C and the reaction was refluxed for 1 h. Reaction progress was monitored by TLC. After completion, the reaction mixture was transferred to a polypropylene tube and was precipitated by adding 1.5 ml 1 M HCl, followed by brief centrifugation. The supernatant was removed and the precipitate was washed with 50% methanol, centrifuged, and the supernatant was discarded. The resulting solid was washed with water and then lyophilized.

### Synthesis of VRK1 fluorescent probes

To each reaction, (Z)-3-(5-(3,5-difluoro-4-hydroxybenzylidene)-2,4-dioxothiazolidin-3-yl)propanoic acid (1.2 eq) and N-hydroxysuccinimide (5 eq) were dissolved in DMF in a glass vial equipped with a stir bar. To the solutions, 1-ethyl-3-(3-dimethylaminopropyl)carbodiimide (5 eq), fluorophore (fluorescein cadaverine - Biotium cat. # 92000, BDP FL amine BroadPharm cat. # BP-23861, or BDP 558/568 amine BroadPharm cat. # BP-28943) (1 eq), and N,N-diisopropylethylamine (10 eq) were added and the reactions were stirred for 30 min at room temperature. The reactions were monitored by HPLC-MS. Purification of products was performed on a Shimadzu LC Nexera Prep HPLC system equipped with a UV-Vis PDA (254, 280 nm) and a Shimadzu Shim-pack GIS C18 reverse phase preparative column (20 μm, 30 × 250 mm). Acetonitrile and water containing 0.5% TFA were used as the mobile phase at a flow rate of 50 ml/min (gradient: 0% to 100% acetonitrile over 35 min, then returned to 0% acetonitrile for 3 min). Purity was assessed by UPLC-MS using a Waters Acquity UPLC system (PDA eλ detector, AcquityQDa mass detector) containing ACQUITY UPLC BEH C18 column (1.7 μm, 2.1 × 100 mm). Acetonitrile and water containing 0.5% formic acid were used as the mobile phase at a flow rate of 0.6 ml/min (gradient: 10% to 100% acetonitrile over 2.8 min, then returned to 10% for 0.2 min). Compounds were identified by mass-to-charge ratio and collected fractions were pooled and lyophilized.

### Data availability

All data supporting the findings of this study are available in the main text or in the supporting information. Source data are available from the corresponding author upon reasonable request. The crystal structure of VRK1 in complex with Compound 1 has been deposited in the PDB under accession code 9ZKG.

## Supporting information

This article contains supporting information.

## Conflicts of interest

The authors declare that they have no conflicts of interests with the contents of this article.
