# Supplementary material for: Structure-based discovery of selective vaccinia-related kinase 1 inhibitors and fluorogenic active-site probes
Source: J Biol Chem. 2026 Mar 6;302(5):111355. doi: 10.1016/j.jbc.2026.111355 (PMC13140042; doi:10.1016/j.jbc.2026.111355)
Supplement: Supporting information [file mmc1.docx]

**Supporting Information**

**Table S1. IC_50_ values of VRK1 inhibitors discovered by initial docking screen. For compounds purchased from MolPort, catalog numbers are listed with “MolPort” prefix. For compounds purchased from Enamine, catalog numbers are listed with “Z” prefix. IC_50_ values were determined with commercial TR-FRET kinase activity assay described in Methods.**

| **Compound** | **IC_50_ (µM)** | **SMILES** | **MolPort/Enamine ID** |
| --- | --- | --- | --- |
| Compound 1 | 0.15 | CCCCN1C(=O)S\C(=C/C2=CC(Cl)=C(O)C(Cl)=C2)C1=O | MolPort-002-238-383 |
| Compound 2 | 10 | NS(=O)(=O)C=1C=CC(=CC1)CNC(=O)C2=CC=C3N=CC=CC3=C2 | Z971205582 |
| Compound 3 | 2.4 | O=C(O)C=1C=CC(=CC1)C(=O)NCC2CCOC3=CC=CC=C32 | Z1603752378 |
| Compound 4 | 1.8 | COC=1C=CC(=CC1Cl)C=2C=CC(=NC2)NC(=O)C3=CC(C)=NO3 | Z1940759774 |
| Compound 5 | 3.7 | COC1=CC=C2C3=C(CCC2=C1)C(=NN3)C(=O)NC1=CC=C(F)C(Cl)=C1 | MolPort-007-849-195 |
| Compound 6 | 6.3 | FC1=CC=C(NC(=O)C2=NNC(=C2)C2=CC=NC=C2)C=C1F | MolPort-010-685-959 |
| Compound 7 | 9.8 | ClC1=CC=CC(=C1)C(=O)NCC1CCN(CC1)C1=CC(=NN1)C1=CC=NC=C1 | MolPort-010-939-476 |
| Compound 8 | 12 | CCC1C=2C=CSC2CCN1C(=O)C=3C=CC(=CC3)C(=O)O | Z927556772 |
| Compound 9 | 14 | COC1=CC=C(C=C1)C(=O)NC1CCCN(C1)C1=CC(=NN1)C1=CC=NC=C1 | MolPort-010-939-463 |

**Table S2. Validation of active site fluorescent probe for accurate K_i_ determination of ATP site inhibitors. VRK-IN-1 activity assay value as published in (41) and VRK1/CK1-IN-1 activity assay value as published in (42). Compounds in which no K_i_ value is reported by probe displacement did not score in the probe displacement assay, suggesting false inhibitors by TR-FRET activity assay, or non-ATP competitive inhibition. *denotes IC_50_ instead of K_i_.**

| **Compound** | **TR-FRET Activity Assay Ki or IC_50_ (µM)** | **Probe-Displacement TR-FRET Ki (µM)** |
| --- | --- | --- |
| VRK-IN-1 | 0.150* | 0.11 |
| VRK1/CK1-IN-1 | 0.0379 | 0.023 |
| Compound 1 | 0.082 | 0.14 |
| Compound 2 | 10* | 3.1 |
| Compound 3 | 2.4* | 21 |
| Compound 5 | 3.7* | Binding not calculated |
| Compound 6 | 6.3* | Binding not calculated |
| Compound 12 | 5.7* | 3.7 |

**Table S3. Data collection statistics of the X-ray diffraction of the crystals of VRK1/Compound 1**

| Space group | P2_1_2_1_2_1_ |
| --- | --- |
| Unit cell (Å) | 91.46 95.93 191.83 |
| Resolution (Å) | 95.92 2.05 (2.18-2.05^a^) |
| Total reflections | 1405558 (227607^a^) |
| Unique reflections | 106167 (16844^a^) |
| Averaged redundancy | 13.2 (13.5^a^) |
| R _meas_^b^ (%) | 26.4 (234.6^a^) |
| CC (1/2) | 99.6 (47.9^a^) |
| Completeness (%) | 99.8 (98.8) |
| I/σ (I) | 9.9 (1.1) |
| R ^c^ (%) R_free_ ^c^ (%) | 18.1  21.6 |
| RMS deviations from ideal values Bond lengths (Å) Bond angles (°) | 0.004  0.643 |
| Ramachandran plot (%) Residues in most favourable regions Residues in accepted regions Residues in disallowed regions | 96.7 3.3 0.0 |
| Clashes | 1.37 |
| Molprobity score | 1.08 |
| Number of molecules (averaged B factor Å^2^)  Protein MP60 Ions, PEGs, buffer molecules H_2_O | 4 (41.7) 2 (65.1) 31 (49.3) 539 (41.0) |

^0^

^a^ Number in parenthesis are for highest resolution shell. redundancy independent R-factor. Calculated for I > -3σ (I). ^c^ . ^d^ R_free_ is equivalent to R_cryst_, but calculated with reflections (5%) omitted from the refinement process. RMS *root-mean-square*. Anomalous correction: percentage of correlation between random half-sets of anomalous intensity differences. Anomalous signal: mean anomalous difference in units of its estimated standard deviation . F(+), F(-) are structure factor estimates obtained from the merged intensity observations in each parity class.


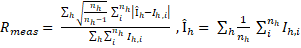

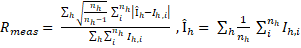

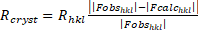

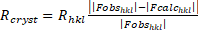

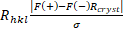

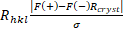


**
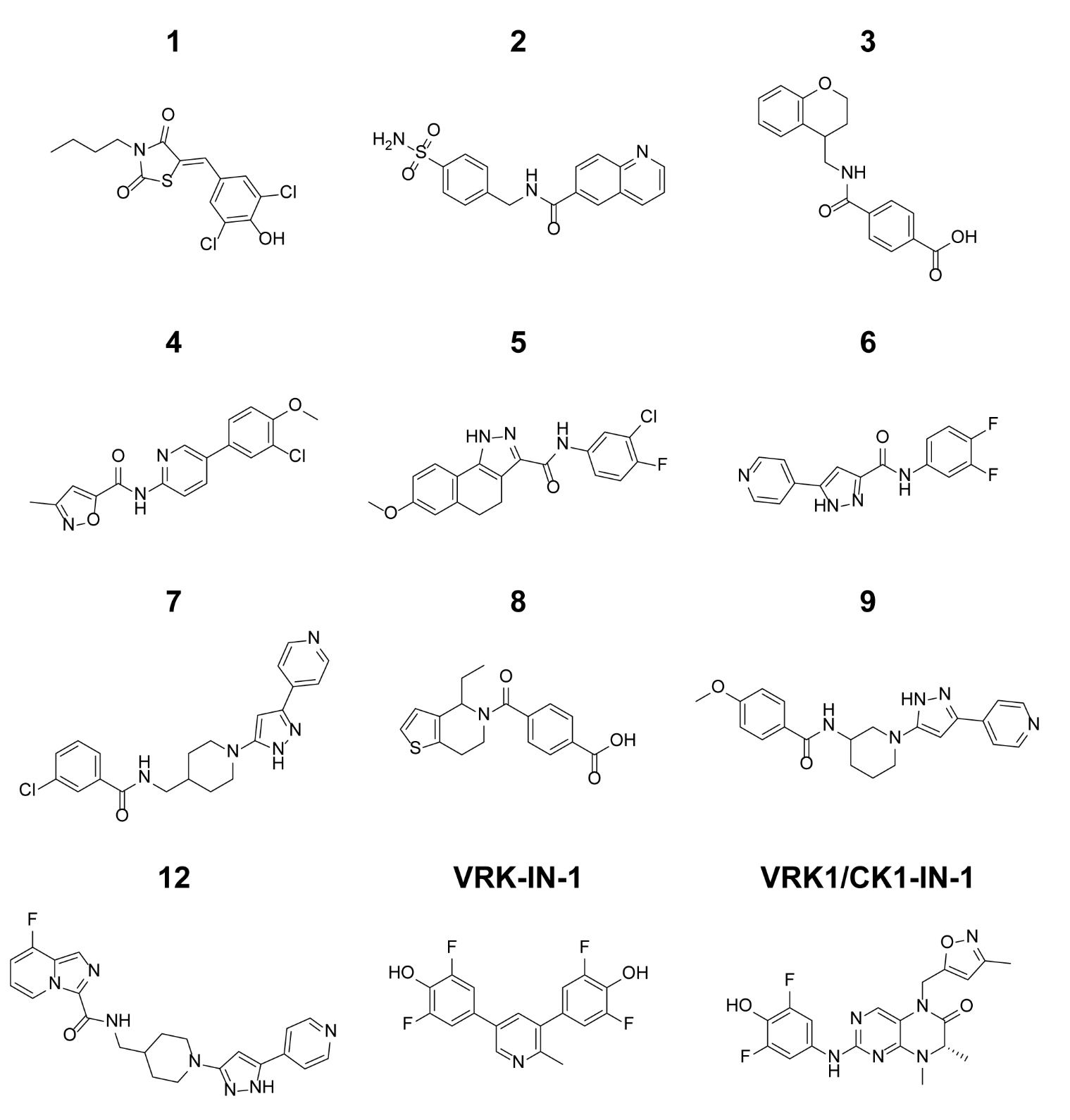
**

**Fig. S1. Structures of compounds described in this work. Compounds 1-9 are from the initial docking screen. Compound 12 was custom-designed after multiple rounds of structure-guided design. Compounds VRK-IN-1 and VRK1/CK1-IN-1 are previously published inhibitors that are now commercially available.**

**
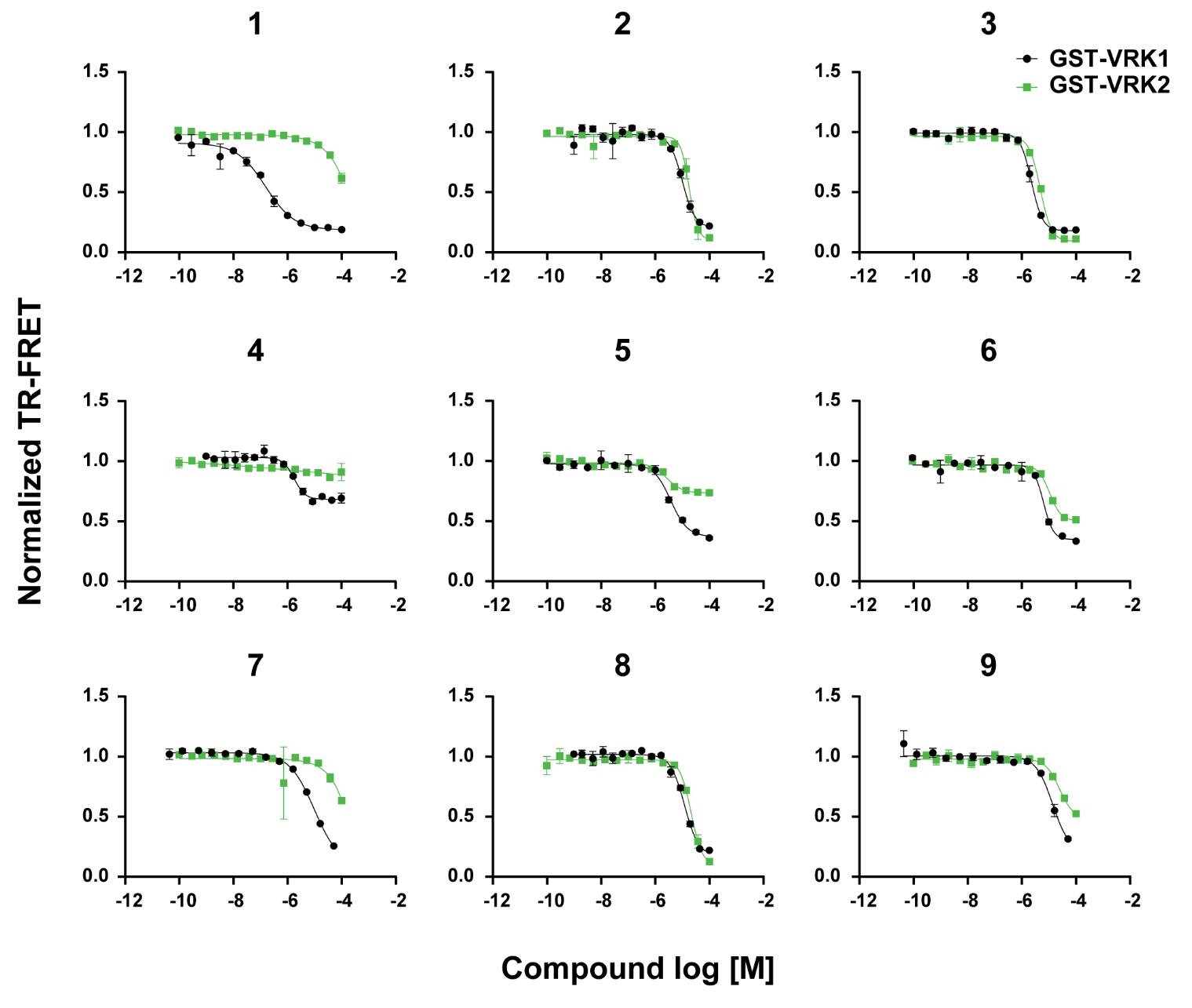
**

**Fig. S2. Dose-response curves using the commercial TR-FRET kinase activity assay of the 9 hits from the initial docking screen.**

**
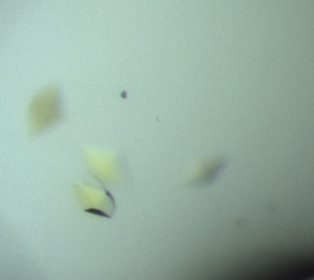
**

**Fig. S3. Crystals of VRK1 with Compound 1.**

**Sequences:**

**GST-VRK1:**

MSPILGYWKIKGLVQPTRLLLEYLEEKYEEHLYERDEGDKWRNKKFELGLEFPNLPYYIDGDVKLTQSMAIIRYIADKHNMLGGCPKERAEISMLEGAVLDIRYGVSRIAYSKDFETLKVDFLSKLPEMLKMFEDRLCHKTYLNGDHVTHPDFMLYDALDVVLYMDPMCLDAFPKLVCFKKRIEAIPQIDKYLKSSKYIAWPLQGWQATFGGGDHPPKSDLVPRGSPENLYFQGHMPRVKAAQAGRQSSAKRHLAEQFAVGEIITDMAKKEWKVGLPIGQGGFGCIYLADMNSSESVGSDAPCVVKVEPSDNGPLFTELKFYQRAAKPEQIQKWIRTRKLKYLGVPKYWGSGLHDKNGKSYRFMIMDRFGSDLQKIYEANAKRFSRKTVLQLSLRILDILEYIHEHEYVHGDIKASNLLLNYKNPDQVYLVDYGLAYRYCPEGVHKEYKEDPKRCHDGTIEFTSIDAHNGVAPSRRGDLEILGYCMIQWLTGHLPWEDNLKDPKYVRDSKIRYRENIASLMDKCFPEKNKPGEIAKYMETVKLLDYTEKPLYENLRDILLQGLKAIGSKDDGKLDLSVVENGGLKAKTITKKRKKEIEESKEPGVEDTEWSNTQTEEAIQTRSRTRKRVQK

**6xHis-GST-VRK2:**

MGSSHHHHHHSSGLVPRGSHMASMTGGQQMGRGSKLMSPILGYWKIKGLVQPTRLLLEYLEEKYEEHLYERDEGDKWRNKKFELGLEFPNLPYYIDGDVKLTQSMAIIRYIADKHNMLGGCPKERAEISMLEGAVLDIRYGVSRIAYSKDFETLKVDFLSKLPEMLKMFEDRLCHKTYLNGDHVTHPDFMLYDALDVVLYMDPMCLDAFPKLVCFKKRIEAIPQIDKYLKSSKYIAWPLQGWQATFGGGDHPPKSDLVPRGSPENLYFQGHPFPEGKVLDDMEGNQWVLGKKIGSGGFGLIYLAFPTNKPEKDARHVVKVEYQENGPLFSELKFYQRVAKKDCIKKWIERKQLDYLGIPLFYGSGLTEFKGRSYRFMVMERLGIDLQKISGQNGTFKKSTVLQLGIRMLDVLEYIHENEYVHGDIKAANLLLGYKNPDQVYLADYGLSYRYCPNGNHKQYQENPRKGHNGTIEFTSLDAHKGVALSRRSDVEILGYCMLRWLCGKLPWEQNLKDPVAVQTAKTNLLDELPQSVLKWAPSGSSCCEIAQFLVCAHSLAYDEKPNYQALKKILNPHGIPLGPLDFSTKGQSINVH

**6xHis-HaloTag-VRK1:**

MGSSHHHHHHSSGLVPRGSHMASMTGGQQMGRGSMAEIGTGFPFDPHYVEVLGERMHYVDVGPRDGTPVLFLHGNPTSSYVWRNIIPHVAPTHRCIAPDLIGMGKSDKPDLGYFFDDHVRFMDAFIEALGLEEVVLVIHDWGSALGFHWAKRNPERVKGIAFMEFIRPIPTWDEWPEFARETFQAFRTTDVGRKLIIDQNVFIEGTLPMGVVRPLTEVEMDHYREPFLNPVDREPLWRFPNELPIAGEPANIVALVEEYMDWLHQSPVPKLLFWGTPGVLIPPAEAARLAKSLPNCKAVDIGPGLNLLQEDNPDLIGSEIARWLSTLEISGSDLVPRGSPENLYFQGHMPRVKAAQAGRQSSAKRHLAEQFAVGEIITDMAKKEWKVGLPIGQGGFGCIYLADMNSSESVGSDAPCVVKVEPSDNGPLFTELKFYQRAAKPEQIQKWIRTRKLKYLGVPKYWGSGLHDKNGKSYRFMIMDRFGSDLQKIYEANAKRFSRKTVLQLSLRILDILEYIHEHEYVHGDIKASNLLLNYKNPDQVYLVDYGLAYRYCPEGVHKEYKEDPKRCHDGTIEFTSIDAHNGVAPSRRGDLEILGYCMIQWLTGHLPWEDNLKDPKYVRDSKIRYRENIASLMDKCFPEKNKPGEIAKYMETVKLLDYTEKPLYENLRDILLQGLKAIGSKDDGKLDLSVVENGGLKAKTITKKRKKEIEESKEPGVEDTEWSNTQTEEAIQTRSRTRKRVQK

**Crystallography Construct VRK1:**

MHHHHHHSSGVDLGTENLYFQSMRVKAAQAGRQSSAKRHLAEQFAVGEIITDMAAAAWKVGLPIGQGGFGCIYLADMNSSESVGSDAPCVVKVEPSDNGPLFTELKFYQRAAKPEQIQKWIRTRKLKYLGVPKYWGSGLHDKNGKSYRFMIMDRFGSDLQKIYEANAKRFSRKTVLQLSLRILDILEYIHEHEYVHGDIKASNLLLNYKNPDQVYLVDYGLAYRYCPEGVHKAYAADPKRCHDGTIEFTSIDAHNGVAPSRRGDLEILGYCMIQWLTGHLPWEDNLKDPKYVRDSKIRYRENIASLMDKCFPAANAPGEIAKYMETVKLLDYTEKPLYENLRDILLQGLKAIGSKDDGKLDLSVVENGGLKAKTITKKRAAEIEE

**Purification of probes:
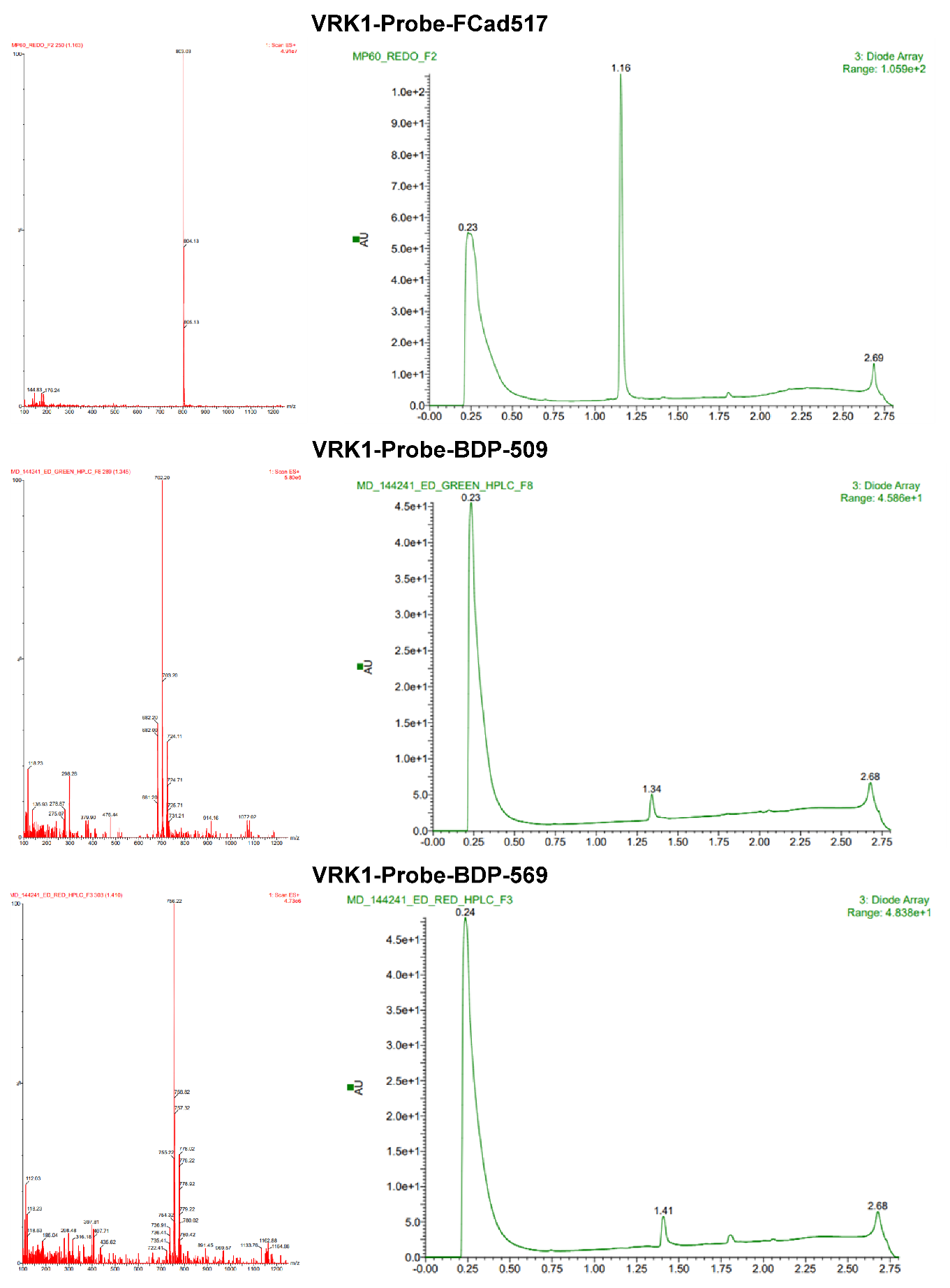
**
